# Adult friendship and wellbeing: A systematic review with practical implications

**DOI:** 10.3389/fpsyg.2023.1059057

**Published:** 2023-01-24

**Authors:** Christos Pezirkianidis, Evangelia Galanaki, Georgia Raftopoulou, Despina Moraitou, Anastassios Stalikas

**Affiliations:** ^1^Lab of Positive Psychology, Department of Psychology, Panteion University of Social and Political Sciences, Athens, Greece; ^2^Lab of Psychology, Department of Primary Education, National and Kapodistrian University of Athens, Athens, Greece; ^3^Lab of Psychology, Section of Cognitive and Experimental Psychology, Department of Psychology, Aristotle University of Thessaloniki, Thessaloniki, Greece; ^4^Lab of Neurodegenerative Diseases, Center for Interdisciplinary Research and Innovation (CIRI - AUTH), Balkan Center, Thessaloniki, Greece

**Keywords:** friendship, wellbeing, adults, PERMA, systematic review

## Abstract

This study aimed to systematically review research findings regarding the relationship between adult friendship and wellbeing. A multidimensional scope for wellbeing and its components with the use of the PERMA theory was adopted. A total of 38 research articles published between 2000 and 2019 were reviewed. In general, adult friendship was found to predict or at least be positively correlated with wellbeing and its components. In particular, the results showed that friendship quality and socializing with friends predict wellbeing levels. In addition, number of friends, their reactions to their friend's attempts of capitalizing positive events, support of friend's autonomy, and efforts to maintain friendship are positively correlated with wellbeing. Efforts to maintain the friendship, friendship quality, personal sense of uniqueness, perceived mattering, satisfaction of basic psychological needs, and subjective vitality mediated this relationship. However, research findings highlighted several gaps and limitations of the existing literature on the relationship between adult friendship and wellbeing components. For example, for particular wellbeing components, findings were non-existent, sparse, contradictory, fragmentary, or for specific populations only. Implications of this review for planning and implementing positive friendship interventions in several contexts, such as school, work, counseling, and society, are discussed.

## 1. Introduction

### 1.1. Adult friendship

Adult friendship is conceptualized as a voluntary, reciprocal, informal, restriction-free, and usually long-lasting close relationship between two unique partners (Wrzus et al., 2017; Fehr and Harasymchuk, 2018).

Mendelson and Aboud (1999) defined six functional components of adult friendship that determine its quality. The first friendship function is *stimulating companionship*, which refers to joint participation in recreational and exciting activities (Fehr and Harasymchuk, 2018). Friends, unlike acquaintances, interact in a more relaxed and carefree way, use more informal language, make jokes, and tease each other (Fehr, 2000).

Existing research literature has mainly focused on the second function of friendship, namely *help* or *social support* (Wallace et al., 2019). Three forms of social support have been identified: (a) *emotional support*, which is conceptualized as acceptance, sympathy, affection, care, love, encouragement, and trust (Li et al., 2014); (b) *instrumental support*, which is defined as provision of financial assistance, material goods, services, or other kinds of help (Nguyen et al., 2016); and (c) *informational support*, which refers to provision of advice, guidance, and useful information (Wood et al., 2015).

The third function of adult friendship is *emotional security*, which refers mainly to the sense of safety offered by friends in new, unprecedented and threatening situations (Fehr and Harasymchuk, 2018). Research has shown that friends can significantly reduce their partners' stress caused by negative life events (Donnellan et al., 2017).

The fourth function of adult friendship is *reliable alliance*, which is defined as the constant availability and mutual expression of loyalty (Wrzus et al., 2017). At the core of reliable alliance lie the concepts of trust and loyalty (Miething et al., 2017).

*Self-validation* is the fifth function of adult friendship. It concerns the individuals' sense that their friends provide them with encouragement and confirmation, thus helping them to maintain a positive self-image (Fehr and Harasymchuk, 2018).

Finally, the sixth function of adult friendship is *intimacy*, which refers to self-disclosure procedures (e.g., the free and honest expression of personal thoughts and feelings; Fehr and Harasymchuk, 2018). It is necessary for both friends to reciprocally reveal “sensitive” information and react positively to the information that their partner discloses to them; in this way, feelings of trust can be developed and consolidated (Hall, 2011).

Adults differ significantly not only with regard to friendship quality, but also to the number of friends one has and the hierarchy of friendships (Demir, 2015). Most individuals maintain small networks of long-term and close friends (Wrzus et al., 2017). Empirical research shows that individuals report an average of three close friends (Christakis and Chalatsis, 2010). Also, individuals make fine distinctions between best, first closest friend, second closest friend, other close friendships, and casual friendships (Demir and Özdemir, 2010). These differentiations reflect the ratings of these friendships regarding several quality indicators (Demir et al., 2011b). The present systematic review of the literature will cover multiple aspects of friendship as predictors of wellbeing, namely friendship quality indicators, number of friends, and friendship ratings.

### 1.2. The concept of wellbeing

Wellbeing is a central issue in the field of positive psychology (Heintzelman, 2018). It is a multifaceted construct (Forgeard et al., 2011) and there are several theoretical approaches of its components (Martela and Sheldon, 2019). We define *wellbeing* as a broad construct that involves the presence of indicators of positive psychological functioning, such as life satisfaction and meaning in life, and the absence of indicators of negative psychological functioning, e.g., negative emotions or psychological symptoms (Houben et al., 2015). This conceptualization captures both *hedonic elements* of wellbeing (Diener, 1984), that involve pleasure, enjoyment, satisfaction, comfort, and painlessness (Huta, 2016) and *eudaimonic elements* (Ryff, 1989), that include concepts like meaning, personal growth, excellence, and authenticity (Huta and Waterman, 2014). Our definition on wellbeing also involves the components of *subjective wellbeing* (Diener et al., 1999), *psychological and social wellbeing* (Ryff, 1989) and *general wellbeing* (Disabato et al., 2019). Finally, this definition encompasses the two different approaches on wellbeing, which are based on the *mental illness model* (Keyes, 2005) and on *positive psychology principles* (Seligman, 2011).

Several attempts have been made to synthesize the aforementioned models. In this systematic review of the literature, we used Seligman's (2011) *PERMA theory* to organize our findings. Seligman (2011) argued that individuals can flourish and thrive if they manage to establish the following five pillars of their lives: positive emotions (P), engaging in daily activities (E), positive relationships (R), a sense of meaning in life (M), and accomplishments (A).

According to the Broaden-and-Build theory (Fredrickson, 2001), when individuals experience *positive emotions*, their repertoire of thoughts and actions broaden (Fredrickson and Branigan, 2005). The broadening effect activates an upward spiral, resulting in the experience of new and deeper positive emotions (Fredrickson and Joiner, 2002). This, in turn, leads to building of resources, which last over time and act as a protective shield against adversity (Tugade and Fredrickson, 2004). Finally, experiencing positive emotions seems to undo the unpleasant effects of experiencing negative emotions (Fredrickson et al., 2000). All the above mechanisms facilitate the physical and psychological wellbeing of individuals (Kok et al., 2013).

*Engagement* describes a positive state of mind in which individuals are fully present psychologically and channel their interest, energy, and time into physical, cognitive, and emotional processes to achieve or create something (Butler and Kern, 2016). Engagement is substantially linked to the experience of *flow*, which is conceptualized as the psychological focus on an activity, accompanied by an experience of high intrinsic motivation and sense of control, and resulting in optimal functioning (Csikszentmihalyi, 2009). High levels of engagement are associated with various indices of wellbeing, such as increased levels of experiencing positive emotions and life satisfaction (Fritz and Avsec, 2007) and decreased levels of anxiety and depression over time (Innstrand et al., 2012).

Positive close *relationships* with family, friends and other significant people are also beneficial. They are found to be associated with emotional and instrumental support, intimacy, trust, increased sense of belonging and other protective indices of physical and psychological wellbeing (Carmichael et al., 2015; Mertika et al., 2020; Mitskidou et al., 2021).

*Meaning* in life reflects the individual's sense that life has coherence, purpose, and significance so that it is worth-living (Martela and Steger, 2016). Research findings show that the presence of meaning in life enhances wellbeing, because it entails high levels of positive emotions and life satisfaction as well as low levels of negative psychological and physical conditions (Pezirkianidis et al., 2016a,b, 2018; Galanakis et al., 2017).

*Accomplishments* in all domains of life are recognized and rewarded by society; this reinforces the individuals' desire to succeed (Nohria et al., 2008). However, accomplishments are not restricted to great achievements in life but also include the fulfillment of daily personal ambitions and the achievement of everyday goals. These minor accomplishments have been found to contribute to the wellbeing of individuals (Butler and Kern, 2016).

### 1.3. Associations between adult relationships and wellbeing components

Positive, supportive relationships predict higher physical and psychological wellbeing levels more than any other variable (Vaillant, 2012). In particular, integrating people into support networks provides them with the necessary resources to successfully deal with depression, anxiety, loneliness, alcohol overdose and many other physical and mental health difficulties (Christakis and Fowler, 2009, 2013). In addition, the chances of individuals' happiness increase when they are associated with a happy person. Therefore, happiness seems to be transmitted through positive relationships (Fowler and Christakis, 2008).

Moreover, research findings show that perceived support from positive relationships is associated with experiencing more positive emotions (Kok et al., 2013), sense of calm and security (Kane et al., 2012), and presence of meaning in life (Hicks and King, 2009).

In particular, adult friendship is a valuable personal relationship (Demir, 2015), which contributes in various ways to individuals' wellbeing (Demir et al., 2007), and significantly fulfills the fundamental human need for social interaction and belonging (Lyubomirsky, 2008). The quality of adult friendship is related to wellbeing and the experiencing of positive emotions (Demir et al., 2007; Secor et al., 2017; Pezirkianidis, 2020). In addition, existing literature shows that a close adult friendship is related to personal achievement and engagement to projects, which promote meaning in life (Green et al., 2001; Koestner et al., 2012).

### 1.4. Gaps in the literature on the relationship between adult friendship and wellbeing

Even though the relationship between friendship and wellbeing has been extensively studied in children (e.g., Holder and Coleman, 2015), adolescents (e.g., Raboteg-Saric and Sakic, 2014), and the elderly (e.g., Park and Roh, 2013), it is not yet fully understood how the various elements of adult friendship are related to wellbeing. The main reason for this is that adulthood consists of many different life periods, from young to late adulthood, during which there are fluctuations in the network of friends and changes in friendship quality (Bowker, 2004).

In fact, most of the empirical research on the relationship between adult friendship and wellbeing focuses on the effect of adult friendship on one-dimensional indices of wellbeing, such as happiness or life satisfaction (Demir et al., 2007). It is worth-noting that research is mainly conducted with university student samples, which limits generalizability to older age groups (Demir, 2015).

### 1.5. The present study

This study aims to systematically review the research literature on the relationship between adult friendship and wellbeing as well as its components, in order to shed more light on the nature of this relationship and the mechanisms that underlie it. More specifically, we will review empirical studies which examined the relationship of quantitative and qualitative indices of adult friendship with wellbeing within the framework of PERMA theory (Seligman, 2011). Therefore, the relationship between adult friendship and overall wellbeing as well as each of its PERMA components will be studied.

Five research questions have been formulated: (a) Which adult friendship variables are mostly associated with wellbeing? (b) Which adult friendship variables predict wellbeing levels based on longitudinal studies? (c) Are there mediating and/or moderating variables in the association between adult friendship and wellbeing? (d) Are there individual differences on the associations between adult friendship and wellbeing? (e) Does adult friendship associate with specific components of wellbeing on the basis of PERMA theory?

## 2. Methods

### 2.1. Criteria of suitability/inclusion of bibliographic sources

We searched for sources reporting empirical research with quantitative and qualitative design using samples ranging in age from 18 to 65 years. Articles were published in scientific journals between 2000 and 2019, since we decided to exclude studies conducted during the COVID-19 pandemic, when the relationships with significant others were negatively affected. We included articles written in English and accompanied by a digital identifier (DOI). Book chapters, reviews and gray literature were excluded.

### 2.2. Search strategy, source selection and data extraction

We searched for resources in the following search engines: Google Scholar, PsycNET, and Scopus. The following algorithm was used to search for the literature sources: “friends” OR “friend” OR “friendship” OR “friendships” AND “wellbeing” OR “wellbeing” OR “psychological wellbeing” OR “psychological wellbeing” OR “happiness” OR “flourish” OR “flourishing” OR “psychological flourish” OR “psychological flourishing” OR “subjective wellbeing” OR “subjective wellbeing” OR “positive emotions” OR “positive emotion” OR “positive affect” OR “engagement” OR “flow” OR “psychological flow” OR “positive relationship” OR “positive relationships” OR “social support” OR “meaning” OR “meaning of life” OR “meaning in life” OR “life meaning” OR “life purpose” OR “purpose of life” OR “purpose in life” OR “achievement” OR “achievements” OR “accomplishment” OR “accomplishments” OR “performance” OR “success” AND “adult” OR “adults”.

The studies were initially selected by two independent evaluators on the basis of their abstract, title and keywords (phase 1). The evaluators were both psychologists and one of them is a researcher, experienced on systematic reviews. The total number of abstracts tested was 1,388. Any paper considered relevant at least by one of the two evaluators was eligible for full-text inspection. The agreement between the evaluators at the first phase was 78%. Thus, 203 articles were included for full-text evaluation (phase 2).

**Figure 1:**
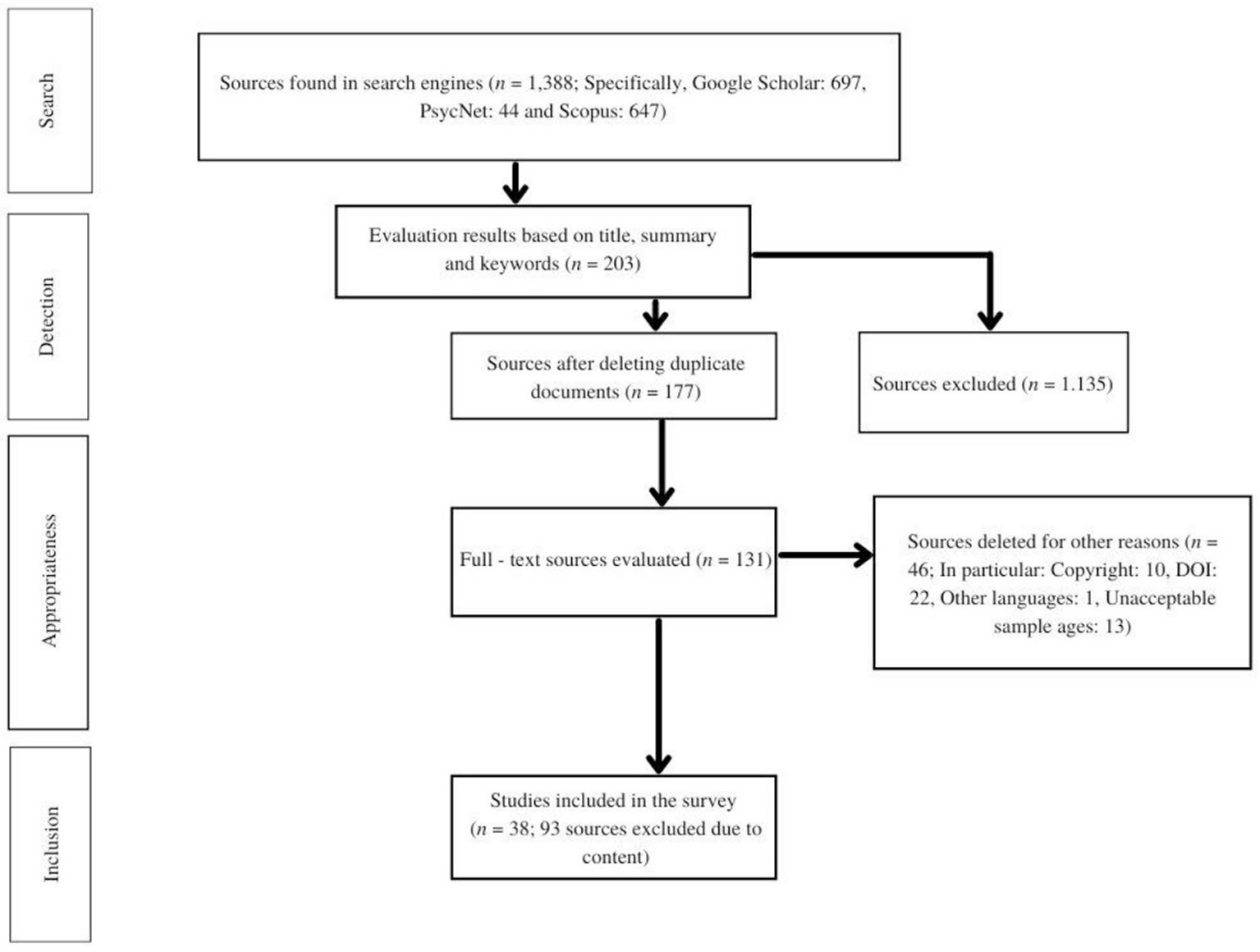
Flow diagram of search strategy and source selection.

During the second phase of the evaluation process, we first checked the sources for duplication and fulfilling the inclusion criteria. The exclusion criteria were the same for both phases. As a result, we removed 33 duplicate documents, 10 articles that their full-text could not be found due to copyright, 22 articles that did not have a DOI, and one article not written in English. In addition, 13 studies were rejected because the sample's age was not within the set limits. Next, the two evaluators independently inspected the full-text of the remaining articles (*n* = 131). As a result, 93 articles were excluded because their content was not relevant to the aims of the study. The agreement between the evaluators at this phase was 96%; if the decision was not unanimous after further discussion, it was excluded by the study. Thus, in total, 38 studies were eligible for the review (see Figure 1).

## 3. Results

From the selected 38 studies, two followed a qualitative design, nine were longitudinal, two had a mixed cross-sectional and experimental design and the rest of them were based on a cross-sectional design. Most of them (*n*=28) used a sample of young adults, mainly university students (see Table 1).

**Table 1:** Findings of the systematic literature review regarding the associations between adult friendship and wellbeing.

**References**	**Study design**	**Sample (*n*, male %, *M_*age*_*)**	**Independent variables**	**Dependent variables (PERMA variable)**	**Measures**	**Key statistical results**
1. Akin and Akin (2015)	CS	USA university students (271, 46%, N/A)	FQ, Subjective vitality	H (WB)	FQS, SHS, SVS	FQ positively correlates with H (*r* = 0.29). Subjective vitality partially mediates this relationship (β = 0.33).
2. Almquist et al. (2014)	Q	Swedish adults born in 1990 (1.289, 50.19%, 19)	FQ, Trust, Self-disclosure	MWB (WB)	Interview *via* phone	Emotional SS, i.e., FQ (*B* = 3.59), trust (*B* = 2.62), and self–disclosure (*B* = 1.61), positively associate with MWB.
3. Brannan et al. (2013)	CS	College students: Iran (151, 59%, 22), Jordan (161, 57%, 21), USA (234, 35%, 25)	SS-Fr	LS, PE (WB, PE)	PSS-Fr, SWLS, PANAS	In USA sample PSS–Fr associates with LS (β = 0.13) and PE (β = 0.26) levels, in the Jordanian sample PSS–Fr associates with PE (β = 0.21) but no significant relationships found in Iran sample.
4. Cable et al. (2012)	L	Adults born in GB in 1958 (6.681, 47.43%, T1: 42, T2: 45, T3: 50)	SNS	PWB (WB)	SNS-SI, Warwicke-Edinburgh MWBS	Smaller friendship networks at age 45 predict lower levels of PWB 5 years later (*B* = −1.30 to −4.72 for less than five friends).
5. Carmichael et al. (2015)	L	USA adults (133, 44.36%, T1: 20, T2: 30, T3: 50)	FQ	PWB (WB)	Social Network Index, FQ-SI, PWB	FQ at 20s predicts FQ at 30s (β = 0.29 to.33), while FQ at 30s predict PWB at 50s (β = 0.38).
6. Carr and Wilder (2016)	CS	USA adults (224, 46%, 21.69)	Risks of seeking SS-Fr	FS, Relational closeness (R)	Risks of seeking social support (5-item scale), Relationship Assessment Scale-FR, Interpersonal Solidarity Scale	Individuals perceiving high risks in seeking social support from friends correlates to lower levels of interpersonal closeness (*r* = −0.38) and friendship satisfaction (*r* = −0.48).
7. Chen et al. (2015)	EXP, CS	University students (study 1: 54 friendship pairs, 24%, 18.56; study 2: 131, 19.85%, 19; study 3: 332, 24.69%, 19)	FQ	SS (R)	Social Support scale, Relationship Quality Scale, Relationship Satisfaction Index	Perceived FQ predicts received support during adversity (β = 0.26) and emotional–focused support among European Americans (β = 0.37). Also, FQ more strongly associates with support provision among European Americans (β = 0.56) than Japanese (β = 0.24), while FQ associates with higher levels of attentiveness (β = 0.42) and companionship (β = 0.38) among Asian Americans than European Americans (β = 0.20 and 0.18, respectively).
8. Cyranowski et al. (2013)	CS	USA adults (692, 43.4%, 43.97)	Companionship with friends	SS, Loneliness, Social distress (R)	UCLA-R, Interpersonal Support Evaluation List, Negative Interaction Scale	Companionship with friends correlates with higher levels of SS from others (r = 0.77) and lower levels of loneliness (r = −0.81) and social distress (−0.27).
9. Demir and Davidson (2013)	CS	USA university students (4.283, 26.38%, 18.81)	PRCA, PM, NS	PE (PE)	PRCAS, MTOQ, PANAS, NS-FR	PM (*r* = 0.32), NS–FR (r = 0.33) and PRCA (*r* = 0.19) positively correlate with PE. NS–FR explains PE levels of men (β = 0.49), while PM (β = 0.09), NS–FR (β = 0.33) and PRCA (β = 0.08) explain PE levels of women.
10. Demir et al. (2007)	CS	USA university students (280, 31.43%, 22.56)	FQ	LS, PE, H (WB, PE)	Network of Relationships Inventory, SWLS, PANAS	The quality of best (*r* = 0.20) and first close friendships (*r* = 0.19) positively correlates with LS and H (*r* = 0.26 and 0.19, respectively), but not with experiencing of PE. Stimulating companionship in best (*r* = 0.29) and first close friendship (*r* = 0.22) associates with H.
11. Demir et al. (2017)	CS	USA university students (2,997, 30%, 19.15)	FQ, PRCA, PNS	H (WB)	MFQ-FF, PRCAS, NSS, SHS, SWLS, PANAS	PRCA and FQ positively correlate to H (*r* = 0.19 to.27 and *r* = 0.26 to.31, respectively). FQ mediates the relationship of PRCA with H in best friendships (β = 0.29 for men and 0.53 for women) and, similarly, PNS in same–sex friendships (β = 0.65 among men and 0.52 amongst women). No differences of gender and same/different–sex friendships were found.
12. Demir et al. (2019)	CS	USA university students (685, 33%, 18.73)	FM, PRCA	SWB, H (WB)	FMS, PRCAS, SWLS, SHS, PANAS	PRCA and FM positively correlates to SWB (*r* = 0.19 and 0.37) and H (*r* = 0.21 and 0.31). FM mediates the relationship of PRCA with SWB (β = 0.11 for men and 0.16 for women) and H (β = 0.08 for men and 0.14 for women). No gender differences found.
13. Demir et al. (2013a)	CS	University students: Turkey (287, 46.69%, 22.22), USA (268, 41.42%, 21.37)	FQ, PRCA	H (WB)	MFQ-FF, PRCAS, SHS	Both in Turkish and Americans FQ and PRCA positively associate with H (*r* = 0.35 and 0.28; *r* = 0.18 and 0.16, respectively). FQ mediates the relationship of PRCA and H in both samples (β = 0.03 for Turkish and 0.04 for Americans).
14. Demir et al. (2012a)	CS	University students: Malaysia (154, N/A, 22.10), USA (211, N/A, 21.95)	FQ	H, Social skills (WB, R)	MFQ-FF, Interpersonal Competence Questionnaire, SHS	FQ both among Americans and Malaysians associates with social skills (β = 0.24 and 0.20) and H (β = 0.33 and 0.38, respectively) and mediates the relationship between social skills and H (β = 0.11 for Americans and 0.15 for Malaysians).
15. Demir and Özdemir (2010)	CS	USA university students (400, 29.25%, 22.39)	FQ, PNS	H (WB)	MFQ-FF, NSS, PANAS	FQ positively correlates with to PNS (*r* = 0.69) and H (*r* = 0.25). PNS mediates the relationship of FQ with H in the three closest friendships (β = 0.26).
16. Demir et al. (2011a)	CS	USA university students (study 1: 256, 32.81%, 20.34; study 2: 498, 21.28%, 19.10; study 3: 299, 20.4%, 19.81, study 4: 175, 30.85%, 20.57)	FAS, FM	H, LS, PE (WB, PE)	FASQ, FMS, SHS, SWLS, PANAS	FAS (*r* = 0.21 to.24) and FM (*r* = 0.41 to.48) positively correlate with H, PE (*r* = 0.18 and 0.43, respectively), and LS (*r* = 0.27 and 0.35, respectively). FM fully mediates the relationship between FAS and H in close and best friendships (β = 0.51).
17. Demir et al. (2011b)	CS	USA university students (study 1: 212, 32.07%, 23.99)	FQ, PM	H (WB)	MFQ-FF, MTOQ, PANAS	PM (*r* = 0.36) and FQ (*r* = 0.21) positively correlate with H. PM mediates the relationship between FQ and H regarding the three closest friendships (β = 0.16 to.21).
18. Demir et al. (2012b)	CS	University students: Turkey (296, N/A, 21.14), USA (273, N/A, 21.80)	FQ, PM	H (WB)	MFQ-FF, MTOQ, PANAS	FQ and PM positively correlate to H among Turkish and Americans (*r* = 0.29 and 0.18; *r* = 0.21 and 0.33, respectively). Among Americans, PM mediates the relationship of FQ and H, whilst among Turkish FQ mediates the relationship of PM with H.
19. Demir et al. (2013b)	CS	USA university students (2,429, 27%, 18.8)	FQ, Sense of uniqueness	H (WB)	MFQ-FF, PSU, PANAS, SWLS, SHS	FQ positively correlates with SoU (*r* = 0.34 to.38) and H (*r* = 0.29 to.32). SoU mediates the relationship between FQ and H (β = 0.38 to.41).
20. Demir and Weitekamp (2007)	CS	USA university students (423, 29.07%, 22.53)	FQ	H, LS, PE (WB, PE)	MFQ-FF, SWLS, PANAS	FQ positively correlates with PE (*r* = 0.25), LS (*r* = 0.18), and H (*r* = 0.26).
21. Derdikman-Eiron et al. (2013)	L	Norwegian adults (1,346, 38.41%, T1: 14.4, T2: 26.9)	Frequency of meeting friends	SS-Fr (R)	Frequency of meeting friends-SI, SS-Fr (2-item scale)	Frequency of meeting friends during adolescence predicts SS–Fr among young adults (OR = 1.33).
22. Griffin et al. (2006)	CS	USA black and white women (290, 0%, 37.8)	SS-Fr satisfaction, Friend network size	LS (WB)	SS questionnaire, LS scale	SS–Fr satisfaction (β = 0.23) and friendship network (β = 0.22) positively associate with LS. No racial differences found.
23. Heck and Fowler (2007)	L	USA secondary and high school students, who became adults seven years later (14.332, 50.9%, N/A)	NF	Participation in community activities (E)	Social network measure, Individual interview	NF of secondary and high school students predicts engagement levels in community activities during young adulthood (β = 0.05).
24. Helliwell and Huang (2013)	CS	Canadian adults (5,025, 49%, 44.93)	NF	LS, H (WB)	NF-SI, Cantril's Self-Anchoring Ladder	NF positively associate with LS and H (β = 0.29 and 0.37, respectively), especially for single, divorced, separated, or widowed individuals.
25. Huxhold et al. (2013)	L	German adults (2.830, 50.8%, 53.3)	SC-Fr	LS, PE (WB, PE)	SC-Fr scale, SWLS, PANAS	SC–Fr positively predicts LS and PE levels 6 years later (β = 0.08 and 0.08).
26. Koestner et al. (2012)	L	105 dyads of friends (210, 0%, 20.19)	FAS	SWB, FQ, Goal progress (WB, R, A)	FQ (5-item scale), SWLS, Goal descriptions and progress ratings	FAS positively correlates with FQ (*r* = 0.60), goal progress (*r* = 0.28), and SWB (*r* = 0.37). FQ positively correlates with SWB (*r* = 0.34). FAS predicts increases in FQ (β = 0.43), SWB (β = 0.21), and goal progress (β = 0.22) 3 months later.
27. Lemay and Clark (2008)	CS	USA (study 1: 96 adults, 15.6%, 34.89; study 2: 86 university students, 38.37%, 21; study 3: 60 pairs of friends, 16.67%, 21; study 4: 96 couples, 50%, 26.5; study 5: 153 adults, 33.33%, 24.63)	Individual's communal responsiveness	SS-Fr, Self-disclosure, Friend's communal responsiveness (R)	Responsiveness (own and friend's), Inventory of Social Supportive Behaviors, SC-Fr-SI, Self-Disclosure Index	Adults' own felt communal responsiveness toward a friend appeared to bias their perceptions of the friend's communal responsiveness (*r* = 0.60), which in turn is associated to own and partner's self–disclosure (*r* = 0.47 and 0.49), evaluation of the friend (*r* = 0.27), and support provision (*r* = 0.40).
28. Li and Kanazawa (2016)	CS	USA adults (15.197, N/A, 21.96)	SC-Fr	LS (WB)	SC-Fr-SI, LS-SI	Frequency of SC–Fr positively associates with LS (β = 0.03), when controlling for marital status.
29. Miething et al. (2016)	L	Swedish adults (772, 50.90%, 23)	Friendship network quality (FNQ)	PWB, FNQ (WB, R)	FNQ-SI, PWB (6-item scale)	FNQ correlates with PWB of young adults both for males and females (*r* = 0.15 and 0.17). FNQ during late adolescence predicts FNQ (β = 0.37 for males and 0.30 for females) and PWB (β = 0.15 and 0.17, respectively) of young adults.
30. Morelli et al. (2015)	Q	49 dyads of same-sex friends (98, 51%, N/A)	Practical and emotional support	SWB (WB)	Personal diaries	Emotional support is associated to wellbeing levels of the actor during time. Practical support is associated to wellbeing of both friends only when the actor is emotionally engaged.
31. Morry and Kito (2009)	CS	USA university students (253, 42.68%, 19.8)	FQ, FS	Relationship supportive behaviors, Relational self (R)	Relational-Interdependent Self-Construal Scale, MFQ-FF-RA, Self-disclosure (10-item scale), Trust (17-item scale), Relationship Assessment Scale, Liking and loving (26-item scale)	FQ and FS positively correlate with relationship supportive behaviors (*r* = 0.76 and 0.75) and the tendency to think oneself in terms of relationships with others (*r* = 0.31 and 0.37).
32. Ratelle et al. (2013)	CS	USA university students (256, 25%, 23)	FAS	SWB (WB)	Learning Climate Questionnaire, SWLS, PANAS	FAS positively correlates with and SWB (*r* = 0.43, β = 0.35).
33. Rubin et al. (2016)	L	AU university students (314, 35.67%, 23.4)	SC-Fr, PS	LS (WB)	SC-Fr-SI, DASS, SWLS	SC–Fr predicts LS 6 months later (β = 0.13).
34. Sanchez et al. (2018)	CS	USA college students (study 1: 273, 30.40%, 19.13; study 2: 368, 32%, 18.90)	FM	H, Compassion (WB, R)	FMS, Compassion Scale, SHS, PANAS	FM correlates with compassion for others and H (*r* = 0.61 and 0.35, respectively) and mediates the relationship of compassion with H (β = 0.18 to.30 for men and 0.24 to 0.29 for women).
35. Secor et al. (2017)	CS, EXP	USA adults (87, 18.39%, 36.87)	SS-Fr, Negative life events	Positive relationships, Life purpose (R, M)	PSSS-Fr, Impact of Event Scale-R, PWBS	SS–Fr positively associates with positive relationships with others and purpose in life after negative life events (*r* = 0.62 and 0.39, β = 0.52 and 0.35, respectively).
36. Walen and Lachman (2000)	CS	USA adults (3.485, 55%, 49.4)	SS-Fr	LS, PE (WB, PE)	Phone interviews, SS-Fr (4-item scale), LS-SI, PE (6-item scale)	SS–Fr positively associate with LS and PE (*r* = 0.23 and 0.22, β = 0.08 and 0.14, respectively).
37. Weiner and Hannum (2013)	CS	USA university students (142, 28.9%, 19.83)	Distance from friends	SS-Fr (R)	Distance status of friends, Inventory of Socially Supportive Behaviors-Modified	Among geographically closer friends received SS positively correlates with perceived emotional (*r* = 0.32), informational (*r* = 0.33) and instrumental support (*r* = 0.23). Closer best friends provide higher levels of perceived and received SS than long distance friends. Received instrumental SS is affected more by long distance from friends (*d* = 0.78).
38. Weisz and Wood (2005)	L	USA university students (80, 50%, N/A)	Social identity support-Fr, Closeness-Fr	FQ (R)	Social Network, Social Support, Social Identity and Social Identity Support Questionnaires	Closeness with and social identity support by another student during the first year predicts best friendship 4 years later (OR = 1.95 and 3.41, respectively).

CS, cross-sectional; EXP, experimental; L, longitudinal; Q, qualitative. T1, first measurement; T2, second measurement. SI, single item. OR, odds ratio. Friendship variables (measures): FAS, friendship autonomy support; FM, friendship maintenance; FQ, friendship quality (MFQ-FF, McGill Friendship Questionnaire-Friendship Functions); FS, friendship satisfaction; NF, number of friends; PM, perceived mattering (MTOQ, Mattering To Others Questionnaire); PNS, psychological needs satisfaction; PRCA, perceived responses to capitalization attempts; SNS, social network size; SC-Fr, social contact with friends; SS-Fr, social support from friends (PSSS-Fr, Perceived Social Support Scale from Friends). Wellbeing indices (measures): A, accomplishments; E, engagement; H, happiness (SHS, Subjective Happiness Scale); M, meaning in life; LS, life satisfaction (SWLS, Satisfaction With Life Scale); PE, positive emotions (PANAS, Positive and Negative Affect Schedule); PWB, psychological wellbeing; R, positive relationships; SWB, subjective wellbeing; WB, wellbeing.

The selected studies were divided into six subgroups on the basis of the PERMA theory of wellbeing and with regard to the associations of friendship with (a) wellbeing, (b) experiencing positive emotions; (c) engagement; (d) building positive relationships; (e) meaning in life; and (f) accomplishments. Also, another analysis was conducted focusing on individual differences regarding the association of friendship variables with wellbeing components.

### 3.1. Associations between adult friendship and wellbeing

Twenty-six studies were found to investigate the association between adult friendship and wellbeing variables. The adult friendship variables studied were friendship quality, best or close friendships, number of friends, support from friends, maintenance of friendship, social interaction with friends and support of autonomy from friends. The wellbeing variables studied were subjective wellbeing, psychological wellbeing, happiness, and life satisfaction. The measures used to measure wellbeing variables were Subjective Happiness Scale (*n* = 9 studies, Lyubomirsky and Lepper, 1999), Satisfaction With Life Scale (*n* = 11, Diener et al., 1985), Positive and Negative Affect Schedule (*n* = 9, Watson et al., 1988), other psychological wellbeing measures (*n* = 5), and single items (*n* = 3; see Table 1).

The results showed that friendship quality significantly associates with wellbeing (Demir and Weitekamp, 2007; Demir et al., 2007, 2011a,b, 2012b, 2013a,b, 2017; Demir and Özdemir, 2010; Akin and Akin, 2015; Carmichael et al., 2015; Miething et al., 2016). In addition, it was found that friendship quality predicts wellbeing levels in the long run. More specifically, friendship quality at the age of 30 predicts wellbeing at the age of 50 (Carmichael et al., 2015). The friendship function, which has been found to mostly correlate with wellbeing levels is stimulating companionship (Demir et al., 2007).

Moreover, perceived emotional or instrumental support offered by friends has been found to significantly associate with wellbeing (Walen and Lachman, 2000; Griffin et al., 2006; Almquist et al., 2014; Morelli et al., 2015; Secor et al., 2017). An interesting finding is that peer support predicts both the provider's and the recipient's wellbeing levels (Morelli et al., 2015).

Regarding socializing with friends, that is, the amount of time individuals spend together, it was found that it also associates with wellbeing levels (Helliwell and Huang, 2013; Huxhold et al., 2013; Li and Kanazawa, 2016), while predicts wellbeing from 6 months to 12 years later (Derdikman-Eiron et al., 2013; Huxhold et al., 2013; Miething et al., 2016; Rubin et al., 2016). Moreover, friends' support of their partners' autonomy (Demir et al., 2011a; Koestner et al., 2012; Ratelle et al., 2013), their reactions to partner's attempts of capitalizing positive experiences (Demir et al., 2013a, 2017), and efforts to maintain the friendship (Demir et al., 2011a) were also found to be positively correlated with wellbeing levels.

Another friendship variable, which was found to be positively associated with wellbeing, is the number of friends (Cable et al., 2012; Helliwell and Huang, 2013). In particular, large and well-integrated friendship networks emerged as a source of wellbeing for adults (Cable et al., 2012). However, no significant associations were found between wellbeing and other friendship variables, such as same gender vs different gender as well as best or close friendships (Demir et al., 2007, 2017).

Finally, six friendship variables were found to mediate the association between adult friendship and wellbeing. These variables are: *maintenance of friendship* (Demir et al., 2011a, 2019; Sanchez et al., 2018), *perceived mattering* (i.e., the psychological tendency to evaluate the self as significant to specific other people, according to Marshall, 2001; see also Demir et al., 2011b, 2012b), *personal sense of uniqueness* (i.e., the tendency to recognize oneself as having distinctive features and to experience worthiness; Demir et al., 2013b), *friendship quality* (Demir et al., 2012b, 2013a, 2017), *satisfaction of basic psychological needs* (Demir and Özdemir, 2010; Demir et al., 2017), and *subjective vitality* (i.e., the conscious experience of possessing energy and aliveness, according to Ryan and Frederick, 1997; see also Akin and Akin, 2015).

### 3.2. Association between adult friendship and PERMA components

#### 3.2.1. Associations between adult friendship and experiencing positive emotions

Seven studies were identified investigating the relationship between adult friendship and experiencing positive emotions (see Table 1). Almost in all studies PANAS (*n* = 6, Watson et al., 1988), was used to measure positive emotions. The results are contradictory regarding the relationship between friendship quality and experiencing of positive emotions. Demir et al. (2007) found no significant relationship, while Demir and Weitekamp (2007) found a low positive correlation. On the other hand, support from friends was found to positively associate with positive emotions among Americans and Jordanians but not Iranians (Walen and Lachman, 2000; Brannan et al., 2013) and predict positive emotions six years later among Germans (Huxhold et al., 2013).

Moreover, research showed that friends' reactions to their partner's attempts of capitalizing positive events, perceived mattering by the friend, psychological needs' satisfaction in friendship (Demir and Davidson, 2013), friend's efforts to maintain the friendship and friendship autonomy support (Demir et al., 2011a) are positively correlated with experiencing of positive emotions. No mediators/moderators of the aforementioned relationships were examined.

#### 3.2.2. Associations between adult friendship and engagement

Only one study was identified investigating the relationship between adult friendship variables and engagement in specific activities (see Table 1). In particular, it was found that the number of friends of secondary and high school students predicts engagement levels in community activities during young adulthood (Heck and Fowler, 2007).

#### 3.2.3. Associations between adult friendship and building positive relationships

Thirteen studies were identified investigating the associations between adult friendship variables and building positive relationships (see Table 1). The results showed that friendship quality and satisfaction positively correlate to relationship supportive behaviors, the tendency to think oneself in terms of relationships with others (Morry and Kito, 2009) and social skills (Demir et al., 2012a). Also, friendship network quality during late adolescence predicts friendship network quality of young adults (Miething et al., 2016). Moreover, friendship quality predicts received support during adversity and emotional-focused support (Chen et al., 2015).

Similarly, companionship with friends during adolescence predicts support from friends during adulthood (Derdikman-Eiron et al., 2013). Also, time spend with friends significantly correlates to higher levels of social support from others and lower levels of loneliness and social distress (Cyranowski et al., 2013). Furthermore, the existing literature reveals an explicit relationship between social support from friends and positive relationships with others (Secor et al., 2017). Taken together, these findings show that adult friendship is an indicator of a well-developed social life.

In addition, support of friends' autonomy is associated with improved quality of friendship after 3 months (Koestner et al., 2012). Individuals who seek support from their friends develop more solidarity-based relationships in their lives, with which they are more satisfied (Carr and Wilder, 2016). Also, received and perceived social support is stronger among geographically closer friends (Weiner and Hannum, 2013) and these friendship maintenance behaviors associate with higher levels of compassion for others (Sanchez et al., 2018). Young adults, especially, build positive, close, supportive and warm relationships if their friends have supported their social identity when they entered university (Weisz and Wood, 2005). Therefore, it is clear that adult friendship exerts a beneficial influence on the quality of concurrent as well as future relationships.

Finally, there is another interesting finding pointing at the mechanisms which lead to positive friendships. When individuals perceive their friends as generous as themselves in their relationship, they are likely to make efforts to maintain and promote the common bond by increasing support and self-disclosure levels in their friendship (Lemay and Clark, 2008).

#### 3.2.4. Associations between adult friendship and meaning in life

Only one study was identified investigating the association between adult friendship variables and sense of meaning in life (see Table 1). In particular, it was found that social support from friends positively associates with purpose in life after negative life events (Secor et al., 2017).

#### 3.2.5. Associations between adult friendship and accomplishments

Similarly, only one study found investigating the relationships between friendship variables and accomplishments (see Table 1). This study found that friendship autonomy support predicts increases in goal progress 3 months later (Koestner et al., 2012).

### 3.3. Individual differences on the relationship between adult friendship variables and wellbeing outcomes

Regarding gender differences, contradictory findings emerged for different friendship variables and their relationship with wellbeing indices. More specifically, perceived mattering by a friend was found to associate with experiencing of positive emotions only among women (Demir and Davidson, 2013), while in the relationship of wellbeing with friend's responses to capitalization attempts, friendship quality and friendship maintenance behaviors no gender differences were found (Demir et al., 2017, 2019). Moreover, no differences were found based on friendship ratings, i.e., between the three closest friendships and their associations with wellbeing indices (Demir et al., 2007; Demir and Özdemir, 2010).

Concerning race, the few studies investigating racial differences focused on comparing Americans with samples from Arabic countries, e.g., Jordan, Malaysia, and Turkey. A few interesting findings focus on the role of support from friends and friendship quality on the wellbeing levels of different samples based on race. More specifically, friendship quality associates more strongly with support provision among European Americans than Japanese, and associates with higher levels of attentiveness and companionship among Asian Americans than European Americans (Chen et al., 2015). On the other hand, Demir and colleagues (Demir et al., 2012a,b) found no racial differences on the relationship between friendship quality and wellbeing among Americans with Malaysians and Turkish. Among Americans, however, perceived mattering by a friend mediates the relationship of friendship quality and wellbeing, whilst among Turkish friendship quality mediates the relationship of perceived mattering with wellbeing (Demir et al., 2012b). Also, as regards the relationship of satisfaction by the support from friends and wellbeing, no racial differences were found among black and white women (Griffin et al., 2006). Nevertheless, support from friend was found to associate with wellbeing in an American sample but not in Jordanian and Iranian samples (Brannan et al., 2013).

## 4. Discussion

The purpose of this study was to systematically review the literature regarding the relationship between adult friendship and wellbeing as well as its components. The existing literature was evaluated through the lens of the PERMA theory (Seligman, 2011), which recognizes five pillars of wellbeing: experiencing positive emotions, engagement, positive relationships, sense of meaning in life, and accomplishments.

The literature review showed that, in general, adult friendship is positively correlated with individuals' wellbeing as well as most of its components. It has been documented that friendship is a valuable personal relationship among adults (Demir, 2015), contributes in various ways to their wellbeing (Pezirkianidis, 2020), enhances their resilience (Mertika et al., 2020; Pezirkianidis, 2020), and fulfills the fundamental human need for social interaction (Lyubomirsky, 2008). However, the instruments used in the previous literature to measure and conceptualize wellbeing significantly vary, i.e., the researchers focus on emotional, psychological, cognitive or subjective aspects of wellbeing making it difficult to draw conclusions and understand the nature of friendships' influences on wellbeing. Also, for particular wellbeing components, the results of the literature review were non-existent, sparse, contradictory or fragmentary, and many were drawn from studies on specific populations.

Concerning the first research question, it was found that the adult friendship variables mostly related to wellbeing are quality of friendship, number of friends, attempts to maintain the friendship, socialization with friends, friends' reactions to partner's attempts to capitalize on positive events, and support from friends (instrumental, emotional or support of autonomy). These findings underlie the importance of studying both qualitative and quantitative dimensions of friendships (Demir and Urberg, 2004; Demir et al., 2007).

As for the second research question, results showed that among the above variables, quality of friendship and socialization with friends predict wellbeing based on longitudinal studies' results. The study of social networks underlines that people's happiness is related to their friends' happiness levels (Fowler and Christakis, 2008; Christakis and Fowler, 2009). Moreover, perceived support from friends, such as companionship, predicts high wellbeing levels more than any other variable (Chau et al., 2010; Forgeard et al., 2011).

In response to the third research question about possible mediators and moderators in the association between adult friendship variables and wellbeing, evidence for moderation was not found. However, six variables were found to mediate this relationship: efforts to maintain the friendship, friendship quality, personal sense of uniqueness, perceived mattering, satisfaction of basic psychological needs, and subjective vitality.

These mediators highlight the possible mechanisms which lead to higher levels of wellbeing. Specifically, when an individual perceives a friend as autonomy supportive, as well as active and constructive responder, *friendship quality* (e.g., intimacy, support, and trust; Demir et al., 2017) and *perceived mattering* increase (Demir et al., 2012b). As a result of these positive friendship experiences, individuals satisfy their *basic psychological needs* (Demir and Özdemir, 2010), realize their *unique* attributes and create a positive self-image (Demir et al., 2013b); therefore, they are likely to engage in *maintenance* behaviors in order to reinforce the resilience and continuity of the friendship (Demir et al., 2011a, 2019). This procedure is enhanced when the individual experiences high levels of energy and *vitality* (Akin and Akin, 2015). Despite the aforementioned findings, further research on mediating and possible moderating effects is clearly needed.

The fourth research question focused on individual differences regarding the associations between adult friendship and wellbeing. The present study found limited differences based on gender and friendship ratings. Previous studies showed significant gender differences concerning friendship functions, but it seems that friendships are equally important for males' and females' wellbeing and prosperity (Christakis and Chalatsis, 2010; Marion et al., 2013). However, significant racial differences were found between samples of completely different cultures, such as Americans and Arabs or Americans and Japanese. More studies needed to shed light on the racial differences between samples of other cultures as well.

The fifth research question focused on whether adult friendship variables can predict specific components of wellbeing on the basis of the PERMA theory (Seligman, 2011). Regarding adult friendship variables and experiencing positive emotions, it was found that friendship quality, support from friends, perceived mattering by friends and satisfaction of the basic psychological needs by a friend significantly and positively associate with experiencing positive emotions. These findings add to the existing knowledge that positive relationships are emotionally rich and a source of great joy for humans (Ryff, 2014). Studies on social networks have shown that positive emotions are “contagious” and are transmitted among friends (Hill et al., 2010; Coviello et al., 2014). Findings about social support show that when friends interact within a positive emotional atmosphere, their experience broadens and this, in turn, activates an upward spiral which evokes even more positive emotions. In this context, partners enrich their interpersonal resources, such as social support, trust, compassion, perceived positive social connections (Kok et al., 2013), and other friendship qualities that are beneficial for physical and mental health (Garland et al., 2010).

According to the PERMA theory, another nuclear component of wellbeing is building positive relationships. The findings of this literature review showed that adult friendship quality and socialization with friends are associated with higher levels of quality and perceived support on every relationship in individuals' lives. Adult friendship is associated with a developed social life, but also with better and more positive relationships. According to Fowler and Christakis (2008), integrating individuals in support networks provides them with the necessary resources to successfully deal with the adverse effects of loneliness. Support from friends, in particular, has been found to lead to higher levels of engagement and satisfaction from different types of relationships, such as romantic and familial ones (Rodrigues et al., 2017). Finally, Weisz and Wood (2005) pointed out that support and appraisal from friends increase satisfaction with friendship as well as its resilience.

Research findings on the relationship of adult friendship with the other three components of wellbeing are limited. Number of friends was found to be related with engagement to community activities, support from friends was found to associate with meaning in life and accomplishments. Relationships with others and the sense of belonging to a network of relationships are one of the main sources of meaning in people's lives (Sørensen et al., 2019; Zhang et al., 2019) and, thus, create a sense of direction in life and intrinsic motivation to set goals and achieve them (Chalofsky and Krishna, 2009; Weinstein et al., 2013).

### 4.1. Gaps and limitations of the existing literature

The research literature on the associations between adult friendship, wellbeing and its components is currently growing but is also characterized by gaps and limitations which need to be addressed.

First, existing literature focuses on the quality of friendship as a whole rather than on its specific characteristics and functions in relation to wellbeing. In addition, only a few studies used a longitudinal design or were conducted with pairs of friends. Existing longitudinal studies do not focus on the effects of friendship, but rather study it only secondarily and often with a single-item measure. To add more, research has focused on the relationship between adult friendship and one-dimensional wellbeing indices, such as happiness and life satisfaction. No attempt has been made to construct a comprehensive theoretical model in order to account for the effects of adult friendship variables on specific components of wellbeing. Furthermore, most studies have been conducted in university student samples, a fact that limits the generalizability of the results to different age groups. The above gaps regarding the association between adult friendship and wellbeing are in accordance with some previous attempts to map this research field (Demir, 2015). In conclusion, future studies should address all these gaps and limitations, not only in the general population but also in various population subgroups and cultural contexts.

### 4.2. Contribution and practical implications of this study

This literature review has clear clinical and social implications. Counselors, psychologists, coaches, social workers, and educators working in clinical, educational, or work settings could utilize the results of this study in order to design interventions for promoting adult friendships. For example, one of the main goals of positive education in childhood and adolescence is to develop skills for building high-quality friendships. Similar efforts could be made in the university context for promoting students' mental health (Bott et al., 2017). In the workplace, building positive relationships and new friendships between employees could be a priority and lead to higher job satisfaction, engagement and productivity (Donaldson et al., 2019). In addition, during counseling or psychotherapy sessions, mental health professionals could use the information provided by this literature review to enhance their clients' supportive environment, experiencing positive emotions and meaning in life and, consequently, strengthen their resilience (Rashid and Baddar, 2019). At the macro level, efforts to build positive friendships and supportive connections between individuals could lead to better and happier citizens, therefore to happier societies (Oishi, 2012).

### 4.3. Conclusions

This study presented a systematic review of research on how adult friendships contribute to wellbeing as well as its components. Although individuals could reap the benefits of friendship from other social sources as well, it became evident that friendship is a special type of relationship, with a unique contribution to wellbeing. As a result, friendships have survived through the years and, in our days, are considered as vital to psychological flourishing (Wrzus et al., 2017). As Anderson and Fowers (2020) have argued, the most significant contribution of friendship to peoples' lives is the initiation and acceleration of the processes from which wellbeing emerges.

## Data availability statement

The raw data supporting the conclusions of this article will be made available by the authors, without undue reservation.

## Author contributions

CP designed the study, conducted the review and the analyses, and wrote the research article. EG wrote and revised the writing of the article. GR wrote parts of the research article. DM revised the writing of the article. AS supervised all stages of the research procedure. All authors contributed to the article and approved the submitted version.
